# Implementation and Preliminary Analysis of FACT-G Quality of Life Questionnaire within an Oncology Survivorship Clinic

**DOI:** 10.7759/cureus.2272

**Published:** 2018-03-05

**Authors:** Reyna Colombo, Deb J Doherty, Christopher M Wilson, Kodie Krzys, Sarah Lange, Hayley Maynes

**Affiliations:** 1 Rehabilitation Services, Beaumont Children's Hospital, Troy; 2 Human Movement Science Department, Oakland University; 3 Physical Therapy, Oakland University

**Keywords:** breast cancer, quality of life, fact-g, well-being, rehabilitation screening

## Abstract

Purpose: To conduct a descriptive analysis of the results from the Functional Assessment of Cancer Therapy-General (FACT-G) quality of life (QOL) questionnaire, describe the outcomes from the FACT-G to drive treatment recommendations within the breast survivorship clinic and to quantify the severity of QOL issues experienced.

Methods: A retrospective analysis utilizing medical records of participants in a breast cancer survivorship clinic. Measurement data included demographics and FACT-G results. Descriptive analysis of demographics and trends in referral recommendations and FACT-G scores was completed.

Results: All 30 participants were females diagnosed with breast cancer of various stages, ages 28 to 81 years. Approximately 1.5 years elapsed between cancer diagnosis and completion of the FACT-G. Participants received surgery (100%), radiation (76%), and chemotherapy and/or hormonal therapy (43%). Results demonstrated that participants reported having a lack of energy (24%) and were bothered by side effects of their treatment (20%). The greatest impact on functional well-being was difficulty sleeping (50%).

Limitations: Decreased ability to generalize the data to breast cancer survivors due to small sample size from one institution and potential referral bias.

Conclusions: Cancer survivors experience QOL issues throughout the continuum of their care, which can result in long-term effects on their physical, functional, social and emotional well-being. QOL is a major focus for cancer survivors and many times determines a survivor’s healthcare decisions. QOL measurements can be utilized at multiple points during survivorship to identify the need for referrals and to guide interventions.

## Introduction

Quality of life (QOL) has recently become a greater focus in cancer rehabilitation and survivorship care [1]. Quality of life and/or health-related quality of life (HRQOL) are both used interchangeably in the oncology literature. According to the National Cancer Institute (NCI), QOL is defined as “The overall enjoyment of life . . . [that] measure aspects of an individual’s sense of well-being and ability to carry out various activities. The Centers for Disease Control and Prevention (CDC), defines HRQOL as “an individual's or a group's perceived physical and mental health over time” [2-3]. Schandelmaier et al. reported that approximately half of cancer treatment protocols included QOL outcomes, however only 20% reported discrete quality of life data [4]. Throughout the cancer journey, it is important to consider the impact that a cancer diagnosis and its treatments can have on one’s HRQOL during the treatment process, as well as when cancer treatments have ceased. Utilizing HRQOL measurements collected from cancer survivors will help to identify the specific dimensions that require action and will provide guidance for determining the appropriate holistic, multidisciplinary care required. It was shown that survivors of the four most common types of cancer (breast, gynecological, prostate, and colorectal) were likely to experience adverse effects related to HRQOL including physical limitations, cognitive limitations, depression/anxiety, sleep problems, fatigue, pain, and sexual dysfunctions [5]. A study completed by Sehlen et al. assessed psychosocial distress, depression, HRQOL, life satisfaction, coping, and social support among cancer survivors undergoing radiotherapy [6]. The study concluded that while all psychosocial variables were significantly associated with survival, HRQOL was the only variable that is able to predict survival (p=0.009) [6]. Furthermore, Heydarnejad et al. found that instead of measuring lipoprotein levels, blood pressure, and electrocardiogram results, cancer survivors made healthcare decisions based on the impact on HRQOL [7]. 

The Functional Assessment of Cancer Therapy-General (FACT-G) is the most commonly used valid and reliable tool to assess QOL in cancer survivors engaged in clinical research [8]. It is a self-report survey questionnaire completed by the patient that quantifies total QOL and four specific domains: physical, social/family, emotional, and functional QOL. The FACT-G can be used to objectively quantify issues in domains that are not routinely screened for in survivors going through cancer treatments. Hamoen et al. reported that the FACT-G questionnaire is highly recommended for assessing cancer-specific HRQOL, receiving the best rating based on its psychometric characteristics [9]. The FACT-G has been suggested for its utility as a screening tool to determine if cancer survivors need additional supportive care to address specific issues identified by their reported QOL scores (total and domain-specific). Another study completed by Taira et al. determined that with time, the FACT-G subscale scores of physical, emotional, and functional well-being all improved while the social well-being score significantly decreased in breast cancer survivors, indicating a need to address this subscale, as well as overall QOL in cancer survivors [10]. This early identification of HRQOL issues in specific domains of the FACT-G that are not consistently addressed in routine care has the potential to positively impact patient satisfaction and survival rates, reduce recurrence, hospitalizations and decrease the other unnecessary costs to the healthcare system.

Purpose

The purpose of this preliminary study was to conduct a descriptive analysis of the results of the FACT-G QOL questionnaire, describe the results of the rehabilitation screening and provide recommendations. A secondary objective of this study was to describe the utilization of the FACT-G questionnaire within a Midwestern hospital system’s survivorship clinic in relation to the survivor’s cancer journey and continuum of care.

## Materials and methods

Design

A retrospective chart review of written and electronic medical records was conducted from a 458-bed hospital (Beaumont Hospital, Troy) with a 42-bed oncology unit and five outpatient physical therapy clinics. Beaumont Hospital, Troy, is a part of Beaumont Health which is a Midwestern healthcare system.

Setting, patients, and measurements

This hospital system services four adjacent counties with a primary service area population of 918,942. In 2014, Beaumont Hospital, Troy, treated approximately 17% of Michigan’s breast cancer cases and 21% of all cancer cases at this hospital system was breast cancer. In 2015, Beaumont Hospital, Troy, initiated a survivorship clinic to assist breast cancer survivors in navigating an established survivorship care plan. The FACT-G was administered to all participants during a survivorship visit at this location as a QOL assessment.

The FACT-G questionnaire uses a basic scoring system to evaluate 27 items on an ordinal scale, using a five-point rating scale ranging from “not at all” to “very much.” The FACT-G has uniformly high reliability and validity coefficients with an average Cronbach's alpha of 0.88 across 78 studies. Test-retest reliability was 0.92 for the FACT-G total scores [11].

**Figure 1:**
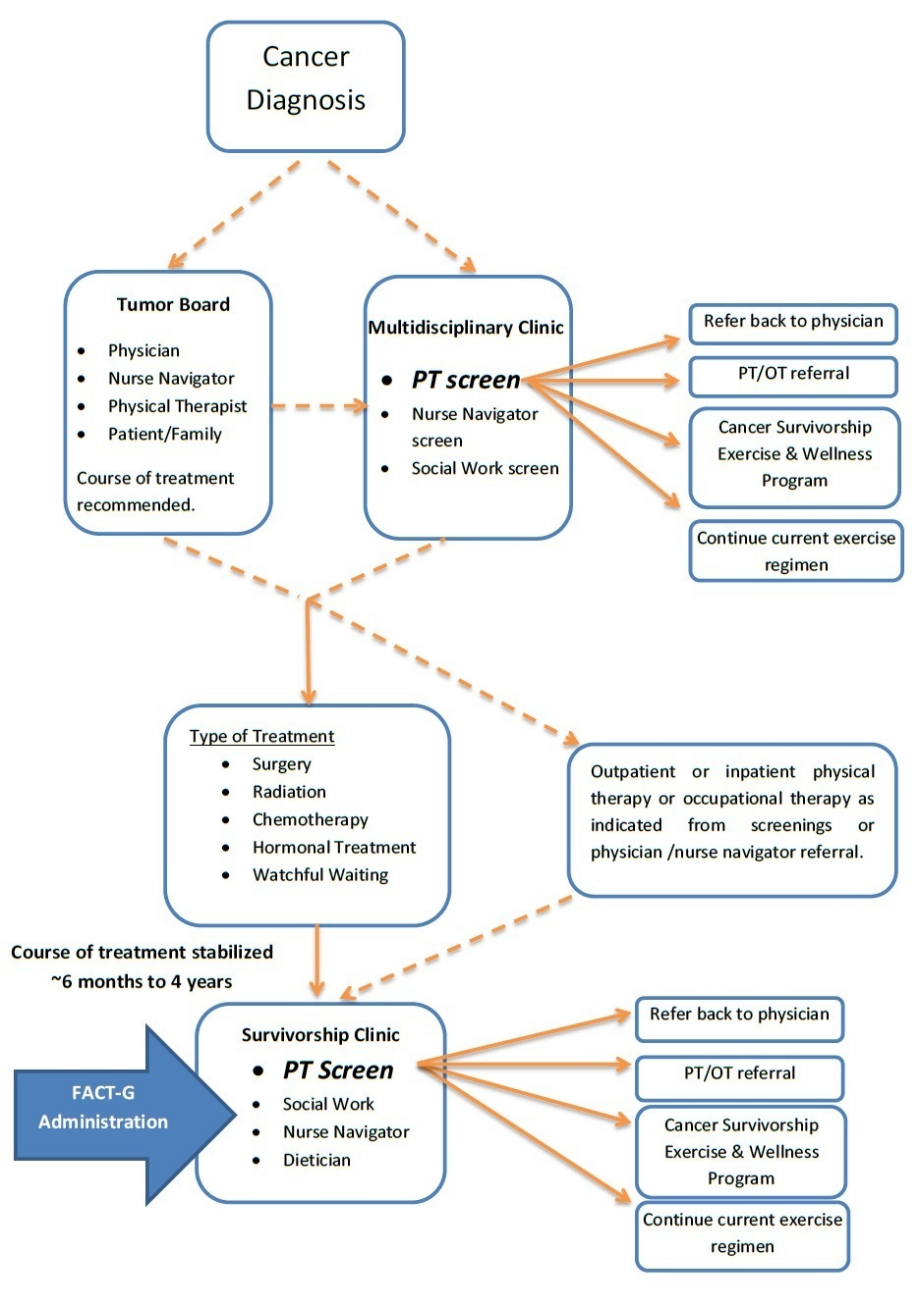
Flow of Cancer Screening

Survivorship visits took place after the participant’s cancer treatment had been completed and served as a component of a survivorship care plan outlined by the Multidisciplinary Clinic (MDC) or by referring physicians (Figure 1). The Breast MDC is an interdisciplinary healthcare panel composed of medical specialists and ancillary team members responsible for creating individualized healthcare plans for referred clients staffed by nurse navigators, physical therapists, nutritionists, and social workers. After the initial diagnosis, the survivor and care team participated in a tumor board to determine a course of treatment. After tumor board was completed, the survivor immediately participated in MDC where she received an initial baseline pre-treatment screening by a physical therapist (PT) with subsequent recommendations. Once the survivor’s treatment was completed or condition was stabilized, she participated in the survivorship clinic. At the survivorship clinic, a FACT-G was completed by the survivor and then provided to the PT for evaluation and determination of forthcoming screening and subsequent recommendations. While in the survivorship clinic, each participant received a one-on-one consultation with an oncology nurse navigator and a PT that lasted for approximately 20 minutes each. In addition, the survivors received a group consultation with a dietician and a social worker. The physical therapy screening was guided by the results of the FACT-G and consisted of a systems review of pain, fatigue, balance, gait, activities of daily living, and a general screen of upper and lower extremity range of motion and strength. In addition, the PT screened for numbness, swelling/edema, dyspnea, insomnia, chemobrain, and other cancer-related symptoms. Based on the results, survivors were referred to either traditional outpatient physical therapy or occupational therapy, a Cancer Survivorship Exercise and Wellness program, instructed to continue the current exercise regimen, or referred back to their physician. (Appendix 1). The Cancer Survivorship Exercise and Wellness program is a self-pay supervised group exercise and wellness clinic administered by a physical therapist assistant with advanced education in cancer rehabilitation and is supervised by a PT.

Intervention

Institutional review board approval was obtained through Oakland University and Beaumont Health System. Electronic and paper medical records of the first 30 consecutive breast cancer survivors who attended Beaumont Hospital, Troy’s survivorship clinic between May 1, 2015, and October 31, 2015, were retrospectively collected and analyzed. Medical records were included for participants who were 18 years or older, had been diagnosed with breast cancer, and had participated in the Breast Survivorship Clinic. Exclusion criteria for this study included those who were present on the Beaumont Health System’s master list of individuals who have requested that their patient care data not be used for research purposes, however, no survivors were excluded based on this criteria. Patient identifiers were removed by a designated hospital employee prior to research team data collection in order to protect patient confidentiality. Data components analyzed included cancer diagnosis, stage of disease, age at diagnosis, age and date at FACT-G distribution, gender, as well as days elapsed from cancer diagnosis and MDC cancer screen to the administration of the FACT-G. The time elapsed between the diagnosis of cancer and the FACT-G responses to surgical, hormonal, radiation, and chemotherapy treatments were also evaluated for relationships.

Analysis

The data was manually inspected for duplicate records and transcription errors. Descriptive statistics, including means and standard deviations, were compiled for subject demographic information. Frequency counts were conducted for the FACT-G subscales and the mean and standard deviations were obtained for the overall FACT-G scores. Inferential statistics were not applicable secondary to the small sample size.

## Results

**Figure 2:**
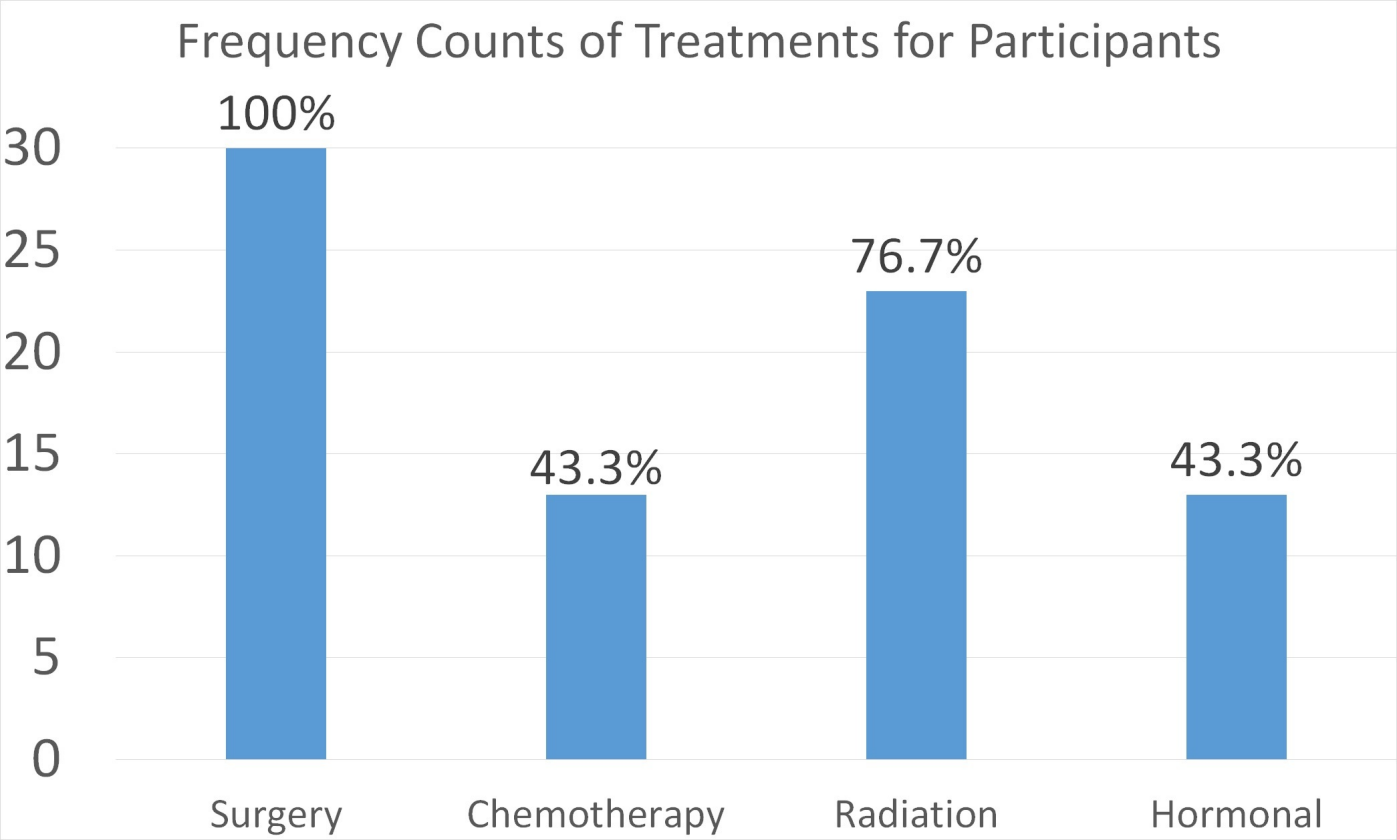
Treatment interventions received

All 30 participants were females diagnosed with breast cancer at various stages (Table 1). The majority of participants had Stage 1 breast cancer (n=16), with Stage 2 being the second most common (n=11). Participants’ ages ranged from 28 to 81 years old at the time of cancer diagnosis (Table 2). The average time elapsed since cancer diagnosis was approximately one-and-a-half years. Figure 2 describes the percentages of survivors who received specific cancer treatments prior to the survivorship clinic. All survivors underwent surgery, while 76% received radiation therapy and approximately 43% of the survivors received chemotherapy and/or hormonal therapy treatment interventions. (Figure 2).

**Table 1:** Participant's Stage of Cancer

Stage of Breast Cancer	Frequency
Stage I	16
Stage II	11
Stage III	2
Stage IV	1

**Table 2:** Survivor Demographics

Statistic Category:	Mean	SD	Min	Max
Age of survivors at cancer diagnosis	62.08	12.88	28.11	81.13
Number of Days (years) from Cancer Diagnosis to FACT-G administered	519.07 (1.42 years)	325.54 (0.89 years)	148 (0.41 years)	1275 (3.49 years)

In the Physical Well-Being subscale (Table 4), survivors reported “quite a bit and very much” to concerns of “having a lack of energy” (24.14%, n=29); “being bothered by the side effects of their treatments” (20%, n=30); and “having pain” (10%, n=30). It is notable that there were no survivors that reported nausea or feeling ill as zero survivors marked that they felt affected “quite a bit” or “very much” for these items. For the Social/Family Well-Being subscale (Table 4), all survivors that reported “quite a bit” or “very much” that their family has accepted their illness and all of the 27 survivors who responded felt that they were close to their partner (or person that is their main support). Results show that 86.67% of survivors felt close to and supported by their friends and 96% felt they receive emotional support from their family and are satisfied with family communication about their illness. Only 17 survivors responded to the item related to satisfaction with their sex life and of those, only 52.94% reported “quite a bit/very much” satisfaction in this area.

**Table 3:** Recommendation following consultation

Therapist Recommendation After Survivorship Visit	Frequency
PT Evaluation	10
OT Evaluation	2
Continue Current Exercise Regimen	9
Cancer Survivorship Exercise and Wellness Program	5
Refer Back to MD	2
No Consultation	2

Table 4 displays the Emotional Well-Being subscale. The results show that 10%-20% of the participants reported feeling sad, nervous, and worried about dying as rated by marking “quite a bit”, or “very much”. Approximately one third (33.33%) of the participants worry “somewhat”, “quite a bit”, or “very much” that their condition will get worse and 16.68% are only somewhat satisfied with how they are coping with their illness.

The Functional Well-Being subscale (Table 4) demonstrates that 90% of survivors have accepted their illness. However, 30% of survivors reported being “not at all”, “a little bit”, or “somewhat” content with their QOL right now. Based on the results, the greatest impact on survivors’ functional well-being has been ‘not sleeping well’ (50%). The majority of survivors reported “quite a bit”/”very much” that they were able to work, that their work was fulfilling, that they were enjoying life, and that they were enjoying the things they usually do for fun.

Following a PT consultation in the survivorship clinic, recommendations were made (Table 3). Two survivors did not receive a consultation due to lack of availability of the PT at the time of the visit. Of the 28 survivors who received consultations, 10 were referred for traditional outpatient physical therapy, two were referred for traditional outpatient occupational therapy, nine were recommended to continue their current exercise regimen, five were referred to the Cancer Survivorship Exercise and Wellness program, and two were referred back to their physician.

**Table 4:** FACT-G responses

Item for Physical Well-Being (n=30 unless otherwise noted)	Quite a Bit/ Very Much	Somewhat	Not at all/ A little bit	Item for Functional Well-Being (n=30 unless otherwise noted)	Quite a Bit/ Very Much	Somewhat	Not at all/ A little bit
I have a lack of energy (n=29)	24.14%	27.58%	48.28%	I am able to work (include work at home) (n=29)	86.21%	10.34%	3.45%
I have nausea	0%	0%	100%	My work (include work at home) is fulfilling	83.34%	13.33%	3.33%
Because of my physical condition, I have trouble meeting the needs of family	3.33%	10%	86.67%	I am able to enjoy life	86.67%	13.33%	0%
I have pain	10%	20%	70%	I have accepted my illness	90%	10%	0%
I am bothered by side effects of treatment	20%	6.67%	73.33%	I am sleeping well	50%	30%	20%
I feel ill	0%	3.33%	96.67%	I am enjoying the things I usually do for fun	73.33%	20%	6.67%
I am forced to spend time in bed	3.33%	3.34%	93.33%	I am content with the quality of my life right now	70.00%	23.33%	6.67%
Item for Emotional Well-Being (n=30 unless otherwise noted)	Quite a Bit/ Very Much	Somewhat	Not at all/ A little bit	Item for Social/Family Well-Being (n=30 unless otherwise noted)	Quite a Bit/ Very Much	Somewhat	Not at all/ A little bit
I feel sad	0%	10%	90%	I feel close to my friends	86.67%	10%	3.33%
I am satisfied with how I am coping with my illness	83.34%	16.66%	0%	I get emotional support from my family	96.67%	3.33%	0%
I am losing hope in the fight against my illness	0%	3.33%	96.67%	I get support from my friends	86.67%	13.33%	0%
I feel nervous	6.67%	13.33%	80%	My family has accepted my illness	100%	0%	0%
I worry about dying	0.00%	20%	80%	I am satisfied with family communication about my illness	96.67%	3.33%	0%
I worry that my condition will get worse	0%	33.33%	66.67%	I feel close to my partner (or the person who is my main support) (n=27)	100%	0%	0%
				I am satisfied with my sex life (n=17)	52.94%	11.77%	35.29%

## Discussion

The purpose of this preliminary study was to conduct a descriptive analysis of the results of the FACT-G QOL questionnaire and describe the results of the rehabilitation screening and recommendations. A secondary purpose of this study was to describe the utilization of the FACT-G questionnaire within a hospital system’s survivorship clinic in relation to the survivor’s cancer journey and continuum of care. The FACT-G questionnaire used in this study has been found to be the most common, valid, and reliable tool to assess QOL in cancer survivors in clinical research [9]. In addition, the FACT-G is highly recommended for cancer-specific HRQOL and received the best rating based on its psychometric characteristics [10].

Of the 28 participants who received a PT screening that was guided by the FACT-G results, the PT identified 43% of the survivors as having met the criteria for referral to traditional physical therapy or occupational therapy services. The PT recommended that 7% of the survivors follow up with their physician for additional medical needs that were outside the scope of physical therapy services. Finally, nine of the 28 subjects (32%) were appropriately exercising at an adequate amount while 17% of participants were referred to the Cancer Survivorship Exercise and Wellness Program for safe progression or introduction of exercises. These clinical findings are relevant as it is unclear as to whether these QOL issues, rehabilitation needs, referrals or exercise interventions would be otherwise administered. This finding highlights the importance of routine evaluation of QOL via a valid, reliable outcome measure, such as the FACT-G. In addition, QOL which was assessed by the FACT-G and interpreted by a PT may assist in the management of physical and functional issues as well as coordinating referrals to other healthcare professionals to optimize the QOL of cancer survivors.

This study adds to the current evidence by demonstrating a documented need for assessing QOL in cancer survivors throughout the continuum of care. Heydarrnejad et al. concluded that cancer survivors made cancer treatment decisions based on their QOL, rather than solely on objective measures such as blood tests [7]. However, the authors of this study suggest that the FACT- G can identify survivors who might need additional healthcare services, such as physical and occupational therapy, social work, or psychology, in addition to those who needed to be referred back to their primary health-care provider. In contrast to previous studies that report about half of cancer treatment protocols included QOL outcomes but only 20% reported specific QOL data [4], the current study objectively quantified QOL issues in domains that are not routinely screened for cancer survivors who have participated in a survivorship clinic.

Harrington et al. found that physical and cognitive limitations, depression, anxiety, sleep problems, fatigue, pain and sexual dysfunctions frequently affect cancer survivors’ QOL and guided their health care choices [5]. Similar to their findings, the results of this study found that decreased sexual satisfaction, sleep quality, and increased fatigue were major contributors to participants’ decreased QOL. This emphasizes the importance of assessing QOL during health care decision-making, which has recently become a greater focus in cancer rehabilitation and survivorship care [1]. Taira et al. found that the social well-being subscale of the FACT-G is the only domain that declines instead of improves one year after cancer surgery, demonstrating that QOL is dynamic and changes over time [8]. This supports the importance of assessing QOL throughout the continuum of care for cancer survivors. Sehlen et al. concluded HRQOL was a major predictor of survival [6]. In comparison, the current study identified the specific domains and aspects of HRQOL that cancer survivors reported to have been affected by their cancer diagnosis and/or treatment. By understanding the impact HRQOL has on cancer survival and combining the information gathered by providing a QOL assessment such as the FACT-G, healthcare professionals can utilize the appropriate tools, approaches, and referrals in order to provide competent and quality care.

The majority of subjects in this study were females over the age of 45 who had been diagnosed with stage one breast cancer. Recently, the American Cancer Society estimated new cases for breast cancer by sex and age groups, projecting that the incidence of cancer diagnosis for those 0-44 years of age, 45-65 years of age, and 65+ years of age to be 11%, 46% and 43% respectively [12]. Comparatively, 7% of the subjects in this study were younger than 45 years of age, 46% between the ages of 45 and 65 years of age, and 47% were 65 years of age and older. These statistics indicate less than a 4% difference between our subjects and the projected national average for these categories, making this study’s data highly generalizable according to age to the American population at large.

Although this study only collected FACT-G data at one point in a cancer survivor’s continuum of care, the findings suggest that QOL issues might be expected to emerge across the entire cancer journey, which shows agreement with previous studies. Each of the 30 participants in this study reported QOL issues, 21 of which required referral to other health-care services. The FACT-G questionnaire identifies the specific QOL dimensions affected by cancer sequelae and can be used to assist health professionals in determining when referrals are needed throughout the continuum of care for cancer survivors.

Objective data and other considerations that impact survivor status should be considered in conjunction with results from standardized instruments, like the FACT- G, to make important healthcare decisions. A variety of healthcare professionals can use QOL tools to establish baseline QOL to assess outcomes, track progress, predict which intervention strategies will facilitate the best results, and assist in referral to interdisciplinary team members.

Study Limitations

Limitations of this study include the utilization of a small, consecutive sample size in only one location in the Midwest. The small sample size of 30 participants, limited the use of multivariate analysis and the generalizability of the results. Bias generated by using data from one institution was also a factor due to geographical location, institutional culture, and referral patterns. These factors could have affected the results and may limit its generalizability. There was also potential referral bias from the physicians depending on their criteria and clinical reasoning utilized to determine which participants required referral to the survivorship clinic. All of these limitations decrease our ability to generalize the data to breast cancer survivors. Data was not available to describe the recommended treatment that patients accepted based on the QOL data nor the outcome of the treatments in terms of improving the QOL or prolonging prognosis. Finally, in order to facilitate system-wide QOL implementation, the FACT- G was chosen as opposed to the FACT- B which specifically assesses breast cancer QOL.

Recommendations for Future Research

To improve on the current study’s design and findings, future studies should include a larger sample size, a QOL assessment throughout the continuum of care including pre-cancer treatment and during or after each treatment (surgery, radiation, chemotherapy). In addition, striving to obtain a more representative sample of total breast cancer incorporate population (including ages, language, nationality, etc.) would increase clinical applicability. It would also be beneficial to collect QOL data from multiple institutions to help improve the generalizability of the data. Further research should be conducted to determine the effects of survivorship programs on QOL for a variety of cancer types, different stages of diagnosis, and multiple recovery periods post diagnosis (i.e. six months, one year, three years). In addition, the predictive validity of the FACT-G should be researched further for its effectiveness in determining the need for referral to specific professional care.

## Conclusions

Cancer survivors experience QOL issues throughout the continuum of cancer care, which can result in long-term effects on physical, functional, social and emotional well-being. This study utilizing the FACT- G QOL questionnaire in a survivorship clinic suggests that QOL measurements taken at multiple points throughout the continuum of care may help identify survivor’s unmet needs that could be addressed through appropriate referrals and guide interventions to improve QOL.

## Appendices

REHABILITATION SERVICES SURVIVORSHIP CLINIC PROGRAM SCREENING

1. Current height:  _________

2. Current weight:  _________

3. Recommendations/Findings:   _________  

         a. Continue current exercise program      

         b. PT Evaluation    

         c. OT Evaluation         

         d. Cancer Survivorship Exercise and Wellness Program  

         e. Other:  _________

4. FACT - G Scores: 

         a. Physical Well Being: _________      

         b. Social/Family Well Being:  _________

         c. Emotional Well Being:      _________

         d. Functional Well Being: _________

5. Pain: _________  /10   Location:   _________ Quality:  _________

6. Current Fatigue Levels (0 = no fatigue / 10 = severe fatigue):  _________

7. Balance: Stumbles/Falls/Near Falls within the last 3 months: Yes    No

8. CURRENT STATUS

          a ADLs (Dressing, self-care homemaking, work, etc)

          b. Gait Distance and speed:

          c. Assistive Device:

          d. Quality of Gait:

9. Symptom Screening:

{ }   Numbness     { } Swelling/Edema   { } SOB  { } Insomnia   { } Chemobrain     { } Other Symptoms: 

10.  Musculoskeletal Tests and Measures

            a. Range of Motion UEs/LEs

            b. Muscle Strength UE/LEs

11. RECOMMENDATIONS AND EDUCATION (see above for additional recommendations)

Specific Interventions provided:

            a. Exercises Provided:

             b Education Provided:

             c. Handouts Provided:
